# Antifungal Compounds against* Candida* Infections from Traditional Chinese Medicine

**DOI:** 10.1155/2017/4614183

**Published:** 2017-12-28

**Authors:** Xin Liu, Zhiming Ma, Jingxiao Zhang, Longfei Yang

**Affiliations:** ^1^Eye Center, The Second Hospital of Jilin University, Changchun 130041, China; ^2^Department of Gastrointestinal Nutrition and Hernia Surgery, The Second Hospital of Jilin University, Changchun 130041, China; ^3^Department of Emergency, The Second Hospital of Jilin University, Changchun 130041, China; ^4^Jilin Provincial Key Laboratory on Molecular and Chemical Genetics, The Second Hospital of Jilin University, Changchun 130041, China

## Abstract

Infections caused by* Candida albicans*, often refractory and with high morbidity and mortality, cause a heavy burden on the public health while the current antifungal drugs are limited and are associated with toxicity and resistance. Many plant-derived molecules including compounds isolated from traditional Chinese medicine (TCM) are reported to have antifungal activity through different targets such as cell membrane, cell wall, mitochondria, and virulence factors. Here, we review the recent progress in the anti-*Candida* compounds from TCM, as well as their antifungal mechanisms. Considering the diverse targets and structures, compounds from TCM might be a potential library for antifungal drug development.

## 1. Introduction

One severe health threat is infections caused by fungal pathogens, among which* Candida* species are the second most common fungal pathogen next to* Cryptococcus neoformans*, responsible for about 400,000 life-threatening infections per annum in the worldwide with a mortality as high as 40% [1, 2].* Candida *spp. accounted for 98% of central venous catheter-related fungemias in patients with cancer [3]. Among the many* Candida* species,* Candida albicans* is the most common fungal pathogen of human diseases, followed by* Candida glabrata*,* Candida parapsilosis*,* Candida tropicalis,* and* Candida krusei* [2].

As the major opportunistic fungal pathogen,* C. albicans* dwells on the skin, in the oral cavity, mucosa of gut, and urogenital tract as a symbiotic fungus under normal conditions [4]. The host could discern the commensal and pathogenic state of* C. albicans*, rendering this fungus under the surveillance of immune system, and the bacterial microbe of locales where* C. albicans* colonize also contributes to keeping this fungus in check [5, 6]. The host defense against* C. albicans* relies on a complicated network consisting of innate and adaptive immune components (e.g., epithelial cells, macrophages, neutrophils, dendritic cells, defensins, and complement). When the hosts encounter lower functions of immune system (resulting from HIV infection, organ transplant, and cancer treatment [7]) or disequilibrium of microflora due to the use of antibiotics [8], mucocutaneous and superficial infections, such as oral thrush and vaginitis, come up. This fungal pathogen could also cause life-threatening systematic infections such as candidemia. Other predisposing factors of* Candida* infections include diabetes and old age [9]. Among the nosocomial bloodstream infections, infections caused by* C. albicans* are the fourth prevalent [10].

In present, the therapeutic drugs for* Candida* infections are limited to five classes of compounds: polyenes, allylamines, azoles, fluoropyrimidines, and echinocandins [11], and amphotericin B, terbinafine, fluconazole, 5-fluorocytosine, and caspofungin are examples for them [12]. Drug resistance emerges due to pervasive application of antifungal drugs, such as fluconazole and voriconazole, for both prophylactic and therapeutic purposes [13]. Cellular and molecular mechanisms underlying drug resistance may include reduced accumulation of intracellular drugs because of increased drug efflux (such as elevated mRNA levels of members of ABC transporter superfamily), mutations in genes of target protein (resulting in elevated levels of target protein or reduced affinity to targets), and modification of metabolism pathways (such as altered synthetic pathway of sterol which plays an important role in both structure and function of fungal cell wall) [14]. Researches indicate extensive regulation of intracellular processes in response to antifungal drugs. The fungistatic property of some drugs such as azoles and 5-flucytosine also contributes to the emergence of resistance [10], while the formation of biofilm may contribute to and elevate the resistance [15]. The paucity of antifungal drugs and the emergence of resistance make it a pressing mission to discover and identify new hits and leads from synthesized chemicals or natural products. Compared to synthesized chemicals, natural products have many advantages such as structural diversity and relatively low toxicity.

Natural products provide a potential source for antifungal drugs, either in their nascent form or as original templates for structure-optimizing for more effective and safe derivatives [16, 17]. Among the marketed antibiotics used clinically, about 80% are derived from natural products [17]. Traditional Chinese medicine is composed of mainly herbs that have been used for thousands of years. Recently, single compounds isolated from many traditional Chinese herbs have been demonstrated to have various kinds of pharmacological activities, such as antibacterial, antitumor, antiviral, and antifungal activities. Considering the present lack of antifungal drugs and the usefulness of traditional Chinese medicine, it may be a promising strategy to develop antifungal agents from traditional Chinese medicines. Here, recent antifungal compounds from traditional Chinese medicines will be briefly reviewed.

## 2. Compounds Targeting Cell Membrane

The plasma membrane keeps the cytoplasm from circumambient environment. The integrity and fluidity of cell membrane means being important to the survival and growth of fungal cells; one important reason is that many enzymes, channels, and transporters of drugs lie on the cell membrane. Cell membrane is the location where many metabolic processes occur and meanwhile it provides a barrier to environmental stresses.

Derived from* Sambucus williamsii*, a traditional herb broadly used for hundreds of years to treat fractures, edema, and scratches in East Asian countries, (−)-olivil-9′-*O*-*β*-D-glucopyranoside exerts its antifungal activity against* C. albicans* by depolarizing the cell membrane evidenced by influx of propidium iodide (PI) and elevated fluorescence of 3,3′-dipropylthiacarbocyanine iodide (DiSC_3_(5), a cyanine dye for measuring membrane potential) [18]. More important and encouraging is that this compound shows little hemolytic activity on human erythrocytes [18]. Two other components (both are lignans) from the same plant, lariciresinol [19] and (+)-pinoresinol, show similar anti-*Candida* effects by damaging the plasma membrane leading to permeabilization [19, 20]. The differential effects upon human and fungi cells imply that it may act on unique components of fungi cells, which needs further identification. Another compound isolated from* Sambucus williamsii*, glochidioboside, shows antifungal activity similar to that of (−)-olivil-9′-*O*-*β*-D-glucopyranoside against* C. albicans* by forming pores on cytoplasmic membrane with a radius range from 1.4 to 2.3 nm [21]. One of the products from the secondary metabolism of* Trachelospermum asiaticum*, dihydrodehydrodiconiferyl alcohol 9′-*O*-*β*-D-glucoside, could also depolarize the transmembrane potential via forming pores with radii ranging from 0.74 nm to 1.4 nm [22]. Changes in granularity and size revealed by the flow cytometry assays also involves alterations of the membrane properties such as osmolarity [22]. However, there are no evident causative link between disruption of membrane potential and changes of osmolarity and no conclusions about which comes first, which remain to be further investigated.

As a component of fungal cell membrane different from the mammalian parallel and a critical modulator for differentiation and pathogenicity of fungi, the glycosphingolipid glycosylceramide in the cell envelope maybe presents a better target for antifungal therapeutic treatments [23].

Ergosterol plays important roles in regulating the fluidity of the cell membrane and cell division of fungal cells, while the structural and conformational differences between ergosterol and sterol (the counterpart of ergosterol in mammalian cells) underlie the antifungal mechanism of the polyenes such as amphotericin B [12, 24]. Despite the low bioavailability and high toxicity of ergosterol-targeting drugs in humans [25, 26], ergosterol still presents a good target for antifungal drugs due to the importance of cell membrane.

Magnolol, one of the major pharmacologically active compounds from* Magnolia officinalis* which could be used to ameliorate the symptoms such as anxiety, asthma, nervous disturbance, and digestive problems [27], could reduce the content of ergosterol in the widely used* C. albicans* SC5314 [28]. Compounds from essential oil of mint, such as menthol, menthone, and carvone, suppress the growth of* C. albicans* through decreasing the contents of ergosterol in cell membrane and the hemolysis caused by them is less than that induced by fluconazole [29]. The ergosterol levels could also be decreased by carvacrol (isolated from* Origanum dictamnus L*.) and thymol, which could exert their influence on the antioxidant defense system, increase the membrane permeability, block the efflux pumps, and thus restore the antifungal susceptibility [30, 31]. Aside from* Candida* species, antifungal activities of this compound against other fungi such as* Monilinia laxa* have been identified [31–33].

Transporters such as ABC transporters on cell membrane could induce the efflux of antifungals, thus compromising the effects of drugs. Treatment with magnolol could significantly decrease the efflux of fluconazole, thus enhancing the antifungal effects of fluconazole [28].

PM-H^+^ ATPase on cell membrane plays a vital role in keeping the transmembrane electrochemical proton gradient which is important for the obtaining of nutrients. The intercellular pH hemostasis modulated by PM-H^+^ ATPase is of great physiological importance. And the enzymatic activity of PM-H^+^ ATPase is positively correlated with cell viability [29]. Carvone, menthol, and menthone could suppress the PM-H^+^ ATPase activity, presumably the primary cause of the antifungal effects [29]. The results also indicated the existence of targets that could be easily touched by external drugs, despite the fact that more efforts need to be made.

## 3. Compounds Targeting Cell Wall Components

The structural integrity of cell wall is vital to the survival and growth of fungal cells, as it provides a shelter from osmotic pressure and other stresses in milieu. Recent studies showed that it likely plays an important role in the colonization and biofilm formation of* C. albicans*, as proteins associated with adhesion, such as Als1, Als3, and Hwp1, are cell wall proteins [34, 35]. Damaged cell wall leads to osmotic fragility of the fungal cell, disrupted membrane, efflux of cytoplasmic contents, and suppressed growth of fungi [13]. Cell wall is lacking in mammal cells, which makes it a preferential target for potential antifungal drugs for safety considerations. The cell wall of* Candida* species holds glycoproteins and abundant carbohydrates, among which are largely glucan, mannose, and chitin [10]. In the following part of this review we will discuss the plant-derived antifungal components acting on cell wall elements or on the synthesis of those elements.

### 3.1. Chitin

As one of the major components comprising fungal cell wall, chitin is a long linear homopolymer of*β*-1,4-linked* N*-acetylglucosamine (GlcNAc) and is synthesized by the incorporation of GlcNAc units from the precursor uridine 5′-diphospho-*N*-acetylglucosamine (UDP-GlcNAc) in a reaction catalyzed by chitin synthetase (CHS) [36, 37].

Despite the small percentage in the cell wall, chitin plays important roles in maintaining the mechanical strength of the fungal cell wall, thus keeping the integrity of the fungal cell wall [38]. Damage to the cell wall may be ameliorated by the elevated quantity of chitin in cell wall due to increased synthesis and/or decreased degradation of chitin, which may increase the tolerance to antifungal drugs [38]. Since this material does not exist in human cells, this presents an attractive target for antifungal therapies [36]. The chemical structures of the classic inhibitors of CHS, namely, polyoxins and nikkomycins, make themselves be degraded easily* in vivo* and difficult to go through the cell membrane, leading to a low antifungal activity [36, 39]. This prompts us to find new CHS inhibitors.

Plagiochin E derived from liverwort* Marchantia polymorpha *L. exerts its antifungal effect through inhibiting the expression of chitin synthetase gene 1 (CHS1) and therefore suppressing the activity of CHS and subsequent synthesis of chitin both* in vivo* and in situ [13]. Interestingly, the expression of CHS2 and CHS3 gene was upregulated by this macrocyclic compound [13]. However, the same group found that plagiochin E exposure of* C. albicans* could induce accumulation of reactive oxygen species (ROS) through malfunction of mitochondria, while pretreatment with L-cysteine could contribute to the survival of* C. albicans* [40]. These studies indicate that plagiochin E may exert its antifungal activity through diverse currently unknown mechanisms.

### 3.2. Glucan

This carbohydrate polymer, together with chitin, is the structural component which holds the integrity and physical strength of the cell wall. The production and assembly of glucan in* C. albicans* need a series of enzymes and regulatory networks, which are fungal-specific and thus render some fascinating targets for antifungal therapies [41]. The most famous drugs of this kind are echinocandins such as caspofungin and micafungin which inhibit the synthesis of*β*-1,3-glucan [10]. Although they are fast-acting, less toxic, and fungicidal [10], mutations in*β*-1,3-glucan synthase that confer resistance to caspofungin have already emerged. Recently, a novel terpene antifungal SCY-078 demonstrated fungicidal activity against* C. albicans* through inhibiting glucan synthase [42]. Sodium houttuyfonate, a derivative from* Houttuynia cordata Thunb.,* might exert its synergistic effect with fluconazole through interfering with*β*-1,3-glucan synthesis and transportation [43].

## 4. Compounds Targeting Mitochondria

The classical respiratory chains of mitochondria are centers of energy production through oxidative phosphorylation, and meanwhile mitochondria are the organelles that produce metabolic intermediates used for amino acid and lipid biosynthesis. Both energy supply and metabolites are indispensable for the survival and growth of* C. albicans*, as well as major cellular event such as yeast-to-hyphal transition. Mitochondria are also involved in efflux-mediated resistance of* C. albicans* to fluconazole [44] while* in vitro* resistance of* C. glabrata* to azoles is associated with mitochondrial DNA deficiency [45]. Resistance to azole is also likely to be related with decreased generation of endogenous ROS that are harmful to DNA, proteins, and lipids while ROS are mainly generated by enzyme complexes (Complex I and Complex III) in classical respiratory chain as by-products of selective degradation of mitochondria [44]. Elevated levels of intracellular ROS are involved in the antifungal effects of fluconazole and miconazole and ROS also play an important role in intrinsic mitochondrial pathway of apoptosis in* C. albicans* [46, 47]. Besides the common enzymes in classical respiratory chain in* C. albicans* cells, there also exist rotenone-insensitive NAD(P)H dehydrogenase and alternative terminal oxidases constituting the cyanide-insensitive respiratory chain [48–50]. In addition,* C. albicans* and* C. parapsilosis* have additional respiratory pathway called parallel respiratory chain [51]. The differences between fungal and mammal mitochondrial enzymes also make developing drugs targeting these enzymes possible [52]. This is the case of some agrichemicals such as boscalid and carboxin that inhibit the succinate dehydrogenase in fungal cells [53]. Although there are only few studies on drugs targeting specifically mitochondria of* Candida *spp., ME1111 [2-(3,5-dimethyl-1*H*-pyrazol-1-yl)-5-methylphenol] did exert its antifungal effects upon human pathogens* Trichophyton mentagrophytes* and* Trichophyton rubrum* through inhibiting succinate dehydrogenase in mitochondria with high selectivity (the IC_50_ values for human cells are more than thirty times higher than that for fungal cells) [53]. In a word, mitochondria might be a promising target for antifungal therapies.

As an important constituent of many herbs of Berberidaceae family such as* Berberis vulgaris*, berberine exerts its antifungal action by induction of mitochondrial dysfunction and increased ROS generation, and its effects are in synergy with fluconazole, even in fluconazole-resistant clinical isolates [54–56]. Moreover, berberine treatment could also culminate in disruption of cell wall integrity in* C. albicans* [57] and inhibit the overexpression of drug resistance gene CDR1 induced by fluphenazine [58]. Although berberine could induce apoptosis in many human cells such as HL-60 leukemia cells and thyroid carcinoma cells [59, 60], berberine could recover the mitochondrial function induced by high-fat feeding in a rat model and could decrease the triglyceride accumulation in the liver in mice [61, 62]. It also markedly decreased the ROS generation in mitochondria [62]. This makes berberine a good candidate for antifungal development although there are much more to be done.

(+)-Medioresinol from the anti-inflammatory, analgesic, and diuretic herbal plant* Sambucus williamsii*, imposed on* C. albicans,* could induce generation of ROS and cell cycle arrest and finally apoptosis [47]. Although (+)-medioresinol could inhibit* in vitro* the proliferation of mammalian cells such as A549, SK-MEL-2, SK-OV-3, and HCT-15 cells at high concentrations, the IC_50_ values for these cell lines were much higher than the MIC value against* C. albicans* cells [47, 63]. In a cohort study in Sweden, (+)-medioresinol in food did not clearly reduce the risk of esophageal and gastric cancers, but at least this compound in diet did not show bad effects [64, 65]. The safe profile of this lignin makes it more inspiring although there is no report on its effects on mammalian mitochondria.

Allyl alcohol from garlic* (Allium sativum),* which has been used as a traditional antimicrobial agent for thousands of years, exerts its antifungal effect through introducing oxidative stress such as increasing ROS production and depleting glutathione. The known targets of allyl alcohol are cytosolic alcohol dehydrogenases Adh1 and Adh2 and the mitochondrial Adh3 [66]. Although allyl alcohol could be released after ingestion of garlic, its toxicity, mediated by acrolein the production of which is catalyzed by alcohol dehydrogenase in rodents, prevents its development as antifungal agent [66, 67].

Baicalin could inhibit the activities of enzymes in mitochondria (such as Ca^2+^-Mg^2+^-ATPase, succinate dehydrogenase, and cytochrome oxidase) and induce cell cycle blockage and apoptosis in* C. albicans* cells [68]. However, in mammalian cells (e.g., CHO cell), baicalin could reduce ROS production [69]. There are also reports showing that baicalin induces apoptosis in human non-small lung cancer cells and osteosarcoma cells through ROS production [70, 71]. Baicalin could protect mitochondria from damage caused by streptozotocin and hepatic ischemia/reperfusion and increase the activity of citrate synthase in rats [72, 73]. Despite the discrepancy between the roles of baicalin in different cells in ROS production, the* in vivo* tests might support the use of baicalin as an antifungal candidate [69, 71, 73].

Shikonin, the major active compound isolated from* Lithospermum erythrorhizon*, could induce the endogenous ROS production, reduce the mitochondrial membrane potential, and alter mitochondrial aerobic aspiration [74]. In human gastric cancer cells and TT medullary thyroid carcinoma cells, shikonin could also induce ROS production and mitochondria-mediated apoptosis [75, 76]. The almost same cytotoxicity for fungal cells and mammalian cells makes shikonin a less attractive candidate for antifungal development.

Curcumin, the yellow pigment isolated from the turmeric (the rhizome of the plant* Curcuma longa Linn*) could also be used as an adjunct drug to treat pathogenic microorganisms such as* Helicobacter pylori*, methicillin-resistant* Staphylococcus aureus* (MRSA), and* Trypanosoma cruzi*. Antifungal activities against various kinds of fungi such as* Candida* species,* Cryptococcus neoformans*,* Aspergillus* spp., and* Sporothrix schenckii* have also been demonstrated by this compound [77, 78]. Curcumin could increase ROS production and apoptosis in* C. albicans* cells, either alone or in synergy with antifungal drugs such as azoles and polyenes [78, 79]. In mammalian cells, curcumin could protect mitochondria from damage and increase the biogenesis of mitochondria, although apoptosis-inducing effects of curcumin have also been reported in cancer cells [80, 81]. Most importantly, this compound could be safe with a maximum tolerance dose of 12,000 mg/day in Phase I clinical trials [82], which present an advantage over other antifungal compounds. However, the poor oral bioavailability and poor solubility in aqueous solutions impede its use and promote the development of methods for delivering curcumin to fight* Candida* infections [83].

Silibinin, the most famous and active compound isolated from* Silybum marianum* (milk thistle) traditionally used to protect from liver injury, could induce apoptosis related to mitochondrial Ca^2+^ influx in* C. albicans* cells [84, 85]. Silibinin could alleviate mitochondrial dysfunction in mice model of cisplatin-induced acute kidney injury through Sirt3 activation, although* in vitro* proapoptotic effects through inducing ROS production have been also reported [86, 87]. The safe profile with silibinin, evidenced by marketed health food and clinical trials, makes silibinin a very promising candidate for antifungal therapies against* Candida* infections although there is still a long way to go [88].

## 5. Virulence Factors

Virulence factors contributing to the* Candida *infections hold adhesins, virulence enzymes (secreted aspartyl proteinase and phospholipases functioning in host tissue invasion) [89], and morphological transition [90].

### 5.1. Yeast-to-Hypha Transition

Although both budding yeast type and hyphal type of* C. albicans* have been found at the loci of infections, the transition of yeast-to-hypha in* C. albicans* is considered to be a major factor involved in the colonization, invasion/penetration, virulence, immune evasion, and survival in the host tissues [91–93]. For instance, most of the hyphal growth of* C. albicans* could not be suppressed by the macrophages after engulfment* in vitro* and lysis of macrophage caused by penetration of hypha was also observed [94]. What is worth mentioning is that, soon after phagocytosis by macrophages, the hyphal formation of* C. albicans* is required (but not sufficient) for inducing the proinflammatory pyroptosis, a kind of programmed cell death of macrophages mediated by inflammasome, before other macrophages are killed by the robust hyphal formation of* C. albicans* [95–97]. Although recently identified Candidalysin secreted by* C. albicans* hyphae plays a vital role in the mucosal pathogenesis through its cytolytic effects, no reports about the relationship between it and macrophages' damage have been published [98]. Nonetheless, Candidalysin renders a promising antifungal target for* C. albicans*. Moreover, hyphae could also support the complicated characteristic structures of mature biofilms which will be discussed later [99]. Induced by exogenous stressors (such as presence of serum and alterations in temperature, pH, levels of oxygen and glucose [91], the presence of* N*-acetyl-*D*-glucosamine (GlcNAc) [100], adherence, and starvation/nutrient limitation [92]), filamentation of* C. albicans* involves underlying alterations in protein synthesis and metabolic changes which are mainly the RAS1-Cyr1p-cAMP-PKA-EFG1 pathway and mitogen-activated protein kinase (MAPK) signaling [91, 92]. Both signaling pathways are governed by the membrane-integrated small GTPase Ras1 [101].

Magnolol and honokiol, two kinds of neolignan isolated from the root, stem, and branch bark of* Magnolia officinalis*, could inhibit the yeast-to-hypha transition of* C. albicans* under many culture conditions. Treatment of magnolol or honokiol could induce downregulation of components of the Ras1-cAMP-Efg1 pathway (such as RAS1, EFG1, TEC1, and CDC35 (the orthologue of Cyr1)), as well as reduced expression levels of the hypha-specific genes ECE1, HWP1, and ALS3, while exogenous cAMP could restore the filamentous growth in the presence of the drugs. These suggest that the transition-inhibiting effects of these two compounds may be associated with the suppression of Ras1-cAMP-EFG1 pathway [102]. Curcumin could also inhibit the yeast-to-hypha transition through targeting the transcriptional suppressor TUP1 (thymidine uptake 1) [79]. Licochalcone-A, a bioactive polyphenol from roots of licorice that has been used as a herbal remedy for hundreds of years, could inhibit the morphological transition [103]. The compound glabridin from licorice and the anthraquinone purpurin from madder root (*Rubia tinctorum* L.) could also inhibit the transition [104, 105].

Apart from the regulating role in antifungal resistance in planktonic* C. albicans*, the chaperone Hsp90 can also modulate the transition by inhibiting the filamentation via cAMP-PKA signaling [106]. Hsp90 deletion in* C. albicans* leads to virulent attenuation in a systemic candidiasis model [107]. So comes the hypothesis that inhibitors of Hsp90 may exhibit anti-*Candida* effects.

### 5.2. Biofilm Formation

Most infections caused by* Candida* spp. involve biofilms formed on the surfaces of biomaterials (such as intravascular catheters and prosthetic heart valves) and biotic mucosa (such as oral cavity and wound surface) [108]. Biofilm, buried in the extracellular matrix (ECM), holds a complicated three-dimensional architecture consisting mainly of yeast form cells and hyphal cells with broad heterogeneity in space [15, 109]. The spatial, structural, and metabolic heterogeneity of biofilms is considered to promote influx of nutrients, efflux of waste products, and establishment of microniches, thus facilitating the adaption of biofilms to the hypoxic environment [99, 110]. Beginning with the adherence of fungal cells to the substrate surface, the development of biofilm undergoes proliferation, maturation, and finally dissemination to finish a cycle and the cycle could repeat itself to expand the fungal population [15]. Cells in the biofilm exhibit great advantages over their free-living parallels in surviving such as increased resistance to many antimycotic drugs (e.g.,* C. albicans* cells of biofilm are almost 1000 times resistant to fluconazole than free-living cells [111]) and protection offered by ECM [15]. The elevated resistance to antimycotic drugs and the ability to withstand host immune defenses, as well as the role as a reservoir for continuing infections, of* Candida* biofilms cause important clinical consequence and the presence of biofilms increases the morbidity and mortality of* C. albicans* relative to strains that could not form biofilms [99, 112]. Therefore, biofilm formation is considered as a potent virulence factor [34]. Now the* Candida* biofilms attract more and more attention, which could be reflected by the increasing number of publications on* Candida* biofilms.

Heat shock proteins play key roles in protecting cells from damage and repairing damage caused by insults, as well as in the protein synthesis, folding, transport and membrane translocation, and so on [9]. Compromising the function of Hsp90 in* C. albicans* by genetic manipulation or pharmacological means could reduce the dispersal and maturation of biofilms as well as increase the sensitivity to drugs used to abolish biofilms [106]. So inhibitors of Hsp90 may present a useful paradigm for therapy of infections caused by biofilm.

In* Candida* cells of biofilms, increased expression of many genes has been found such as genes involved in protein synthesis, drug transporting, adherence to matrix, and primary metabolism [109]. Genes encoding envelope proteins such as Hwp1, Als1, Als3, and Sun41 play critical roles in biofilm formation [92].

Although the structures of* C. albicans* biofilm can be disrupted by physical means such as mechanical removal by brushing on the surface of teeth and ultrasound (or sonication) treatment of implants [113], the clearance of* C. albicans* is primarily dependent on drugs which could prevent the formation of biofilm or abolish the matured biofilm.

Biofilm formation of* Candida *spp. and other fungi could be replicated in 96-well microtiter plates [36] as well as in animal paradigms [15], which provide us with useful tools to screen potential antifungal hits. Derived from* Cinnamomum zeylanicum*, cinnamon oil exhibits antifungal activity against* C. orthopsilosis* and* C. parapsilosis* through inhibiting the formation of biofilm as well as the growth of planktonic counterparts [114], although the exact mechanism is unknown. One of the major components cinnamaldehyde (of the oil) could also inhibit the biofilm formation of clinical isolates of* C. albicans* [115], and moreover it could suppress the growth of* Aspergillus flavus *and* Aspergillus oryzae* which are culprits of food spoilage [116]. Berberine, an alkaloid from the medicinal plants such as* Coptis chinensis* and* Hydrastis canadensis, *also has antifungal activities against* C. albicans* biofilms, both alone and in synergy with miconazole [117]. Licochalcone-A also demonstrated* in vitro* and* in vivo* antifungal activity against* C. albicans* biofilms [103]. Purpurin also demonstrated antifungal activity against the formation and preformed biofilms of* C. albicans*, in addition to its capability of inhibiting morphological transition [104].

Aside from inhibiting the yeast-to-hypha transition, magnolol and honokiol also inhibit biofilm formation via suppressing adhesion and growth of* C. albicans* as is evidenced by XTT assay and confocal laser scanning microscopy. These two compounds could reduce the fungal burden and prolong the lifespan of* Caenorhabditis elegans* in a nematodes infection model [102]. What is more important, compounds at the concentrations used exhibit no adverse effect on the mammalian HSC-T6 cells and nematodes [102]. Curcumin could also inhibit the biofilm formation of* C. albicans* [118]. Thymol (5-methyl-2-(1-methylethyl) phenol), a major essential oil in the herb thyme (*Thymus vulgaris* L., Lamiaceae) which could be applied for treating multiple symptoms including bronchitis, whooping cough, and catarrh of the upper respiratory tract [119, 120], exhibits antifungal activity against fluconazole-sensitive and fluconazole-resistant isolates of* C. albicans* [121]. Recent study identified that thymol could inhibit the biofilm formation and development, and moreover this compound could enhance the host antimicrobial responses against* C. albicans* and increase the lifespan of* C. elegans *during the fungal infection [115, 122]. In addition, thymol has shown synergy with fluconazole against biofilms [115, 123]. Baicalein and aucubin from* Plantago major* (greater Plantain), a perennial herb used for wound healing, analgesic, anti-inflammatory, antioxidant, and infections, could inhibit the biofilm formation and decrease the cell surface hydrophobicity of* C. albicans* [124]. Eugenol, the major components of essential oils from* Syzygium aromaticum* (clove), possesses the capacity to inhibit the biofilm formation and preformed biofilms, more effective than marketed antifungal drug fluconazole. This compound could also produce synergistic effects with fluconazole [115, 123] and what is more, the structure-activity relationship of this compound is analyzed [125]. Another phenylpropanoid from clove, methyleugenol, also exhibits antifungal effect against fluconazole-resistant* Candida* isolates and synergistic effect with fluconazole [126]. Antibiofilm activity of menthol from mint, either alone or in combination with fluconazole, was also identified [123, 127]. So is the case with geraniol (3,7-dimethylocta-*trans*-2,6-dien-1-ol) [115], an acyclic monoterpene alcohol which could be isolated from many herbs such as* Pelargonium graveolens* (Geraniaceae), nutmeg, and ginger [128, 129]. Another compound from* Pelargonium graveolens, *linalool, also exhibits antifungal effect on the planktonic and biofilm cells of* C. tropicalis* [129]. Carvacrol could also sensitize the* Candida* biofilms, as well as the planktonic cells, to fluconazole [130]. Usnic acid (2,6-diacetyl-7,9-dihydroxy-8,9b-dimethyl-1,3(2H, 9bH)-dibenzo-furandione), the major active component isolated from medicinal lichens such as* Cladonia* and* Usnea* [131], could inhibit the formation of* Candida* biofilms and other virulent traits [132, 133]. Berberine, from* Berberis aquifolium, Hydrastis canadensis, Phellodendron amurense*, has the antifungal activities against fluconazole-resistant* Candida* spp. in planktonic and biofilm form [134]. Emodin from rhizomes of* Rheum palmatum* could inhibit the formation of biofilms and hyphal development of* C. albicans* [135].

### 5.3. Other Factors

Virulence in mice caused by* C. albicans* mutants deficient in isocitrate lyase 1 (ICL1, a major component of the glyoxylate cycle) is evidently less than the wide type equivalents, which indicates the involvement of the glyoxylate cycle in the pathogenesis of candidiasis [136]. ICL1, as well as malate synthase, is the distinctive enzyme that has not been observed in mammalian cells; thus it may render a unique target for inhibiting the virulence of* C. albicans* to combat this fungal pathogen. Recently, inhibitors of malate synthase demonstrated antifungal effect against* Paracoccidioides species* [137], while apigenin, the active flavone compound in Chinese herbs such as thyme, could inhibit the enzymatic activity of ICL1 in* C. albicans* [138, 139]. Rosmarinic acid, the bioactive polyphenol in herbs such as basil* (Ocimum basilicum)*, oregano* (Origanum vulgare)*, sage* (Salvia officinalis),* and* Melissa officinalis*, has also been identified as an inhibitor of ICL1 in* C. albicans* [138, 140]. Recent study by Ansari et al. demonstrated that both the enzymatic activity and mRNA expression of ICL1 and malate synthase of* C. albicans* could be inhibited by the monoterpenoid perillyl alcohol, the active compound from edible and medicinal plant* Perilla frutescens* L. ex B. D. Jacks. (Lamiaceae) which has been used for treating colds, food allergy, and depression [141, 142]. Considering the absence of ICL1 in human, the well-tolerated profile in human, and the fact that Phase II trials have been conducted in patients with cancers, perillyl alcohol might serve as an interesting candidate for antifungal therapies against* C. albicans* [143, 144].

Similar to* Pseudomonas aeruginosa*, communication among fungal cells is often associated with virulence [145]. Quorum sensing means that molecules secreted by the* C. albicans* cells in response to cell density could affect the behaviors of the cells. The formation of biofilms, hyphal growth, and virulence factors of* C. albicans* could also be regulated by quorum sensing [146]. The most famous quorum sensing molecule of* C. albicans* is farnesol, one autoregulatory sesquiterpene alcohol that could prevent the filamentation (through repressing the Ras1-cAMP-PKA signaling pathway [147]), shrink the biofilm (if added before attachment or after formation but not during the initial stages of biofilm growth [148]), and block other virulence factors [149]. So comes the strategy that targeting quorum sensing molecules may contribute to the antifungal therapies [146]. Indeed, the dietary flavonoid quercetin isolated from edible and medicinal lichen* Usnea longissimi* could sensitize fluconazole-resistant isolate NBC099 to fluconazole and this kind of sensitization could be the quercetin-induced production of farnesol [146].

Another quorum sensing molecule produced by* C. albicans*, tyrosol, could also affect the development of* Candida* biofilms [150]. This aromatic alcohol could induce the morphological transition from yeast to hyphae. At high concentrations (above 200 mM), tyrosol could cause reduction in biofilms formed by* Candida* species as well as those by* Streptococcus mutans* [151].

Extracellular hydrolytic enzymes produced by* C. albicans* are considered as virulence factors liable for the penetration into and damage to host cells caused by this pathogenic fungus [152]. These enzymes include secreted aspartic proteinases, lipases, and hemolysins [34]. Quercetin could inhibit the activities of proteinase, esterase, phospholipase, and hemolysins of fluconazole-resistant* C. albicans* strain NBC099 [146]. In addition, this compound could also synergize with fluconazole against biofilm both* in vivo* and* in vitro* [153].

## 6. Compounds without Identified Mechanism

Anofinic acid and fomannoxin acid isolated from* Gentiana Algida* showed weak antifungal activities against* C. albicans*, while the esterification by introducing methyl group into those compounds could enhance the anti-*Candida* activities but decrease the activities against the* Cladosporium cucumerinum*, which is a kind of plant pathogenic fungus [154]. However, no further research about the antifungal mechanism has been performed since that finding. Anofinic acid could also be isolated from another traditional Chinese medicine,* Gentiana macrophylla*, which has been used for long as therapies for constipation, pains, jaundice, and rheumatism [155]. Another dihydroflavone isolated from* Gentiana macrophylla*, kurarinone, could also inhibit the growth of* C. albicans* [155]. Nyasol ((Z)-1,3-bis(4-hydroxyphenyl)-1,4-pentadiene), isolated from the herbal plant* Anemarrhena asphodeloides Bunge* (Liliaceae) which has been used in Chinese traditional medicine as antipyretic, anti-inflammatory, antidiabetic, and antidepressant agent [156], exhibits antifungal activity against* C. albicans* alone or in synergy with azoles [157, 158]. This compound also has activity against other fungal pathogens such as* A. flavus*,* Fusarium oxysporum*,* Pythium ultimum,* and* Rhizoctonia solani*, to name a few [158, 159]. Another compound from clove, isoeugenol, also exhibited antifungal activities against* C. albicans*, as well as* Aspergillus niger* [125].*α*-Terpineol (2-(4-methyl-1-cyclohex-3-enyl) propan-2-ol), from* Artemisia annua*, could inhibit a series of* Candida* species isolated from denture stomatitis patients [160]. The sesquiterpene lactone isolated from* Inula racemosa* showed good antifungal activity against* Candida* species, as well as other human fungal pathogens such as* A. flavus* and* Geotrichum candidum* [161].

## 7. Conclusion

In summary, many natural compounds from TCM could exert their anti-*Candida* activities through different mechanism, providing a big reservoir for developing antifungal therapies.

Combination therapies are capable of increasing the efficacy and preventing the emergence of drug resistance and many approaches have been adopted to identify effective combinations, especially the synergistic effects with marketed drugs [162–164]. An important part of the adjuvants to antibiotics might come from the previously undervalued part of chemical entities, which have been recently termed as dark chemical matter (DCM) [165]. Because these DCM showed little or no bioactivity in previous researches towards human targets, it may represent a novel and valuable repertoire for identifying hits and optimizing leads [165]. The machine learning-based synergism prediction may be a promising method to identify synergistic effects of marketed antifungal drugs and natural products isolated from traditional Chinses medicine [162]. Considering new proteins or biological processes that might be used as emerging targets such as histone deacetylase and ion homeostasis, compounds from TCM might play increasing important and diverse roles in developing antifungal therapies against* C. albicans*.
